# Membrane Interactions of Phytochemicals as Their Molecular Mechanism Applicable to the Discovery of Drug Leads from Plants

**DOI:** 10.3390/molecules201018923

**Published:** 2015-10-16

**Authors:** Hironori Tsuchiya

**Affiliations:** Department of Dental Basic Education, Asahi University School of Dentistry, 1851 Hozumi, Mizuho, Gifu 501-0296, Japan; E-Mail: hiro@dent.asahi-u.ac.jp; Tel./Fax: +81-58-329-1266

**Keywords:** phytochemical, molecular mechanism, membrane interaction, membrane fluidity, drug lead

## Abstract

In addition to interacting with functional proteins such as receptors, ion channels, and enzymes, a variety of drugs mechanistically act on membrane lipids to change the physicochemical properties of biomembranes as reported for anesthetic, adrenergic, cholinergic, non-steroidal anti-inflammatory, analgesic, antitumor, antiplatelet, antimicrobial, and antioxidant drugs. As well as these membrane-acting drugs, bioactive plant components, phytochemicals, with amphiphilic or hydrophobic structures, are presumed to interact with biological membranes and biomimetic membranes prepared with phospholipids and cholesterol, resulting in the modification of membrane fluidity, microviscosity, order, elasticity, and permeability with the potencies being consistent with their pharmacological effects. A novel mechanistic point of view of phytochemicals would lead to a better understanding of their bioactivities, an insight into their medicinal benefits, and a strategic implication for discovering drug leads from plants. This article reviews the membrane interactions of different classes of phytochemicals by highlighting their induced changes in membrane property. The phytochemicals to be reviewed include membrane-interactive flavonoids, terpenoids, stilbenoids, capsaicinoids, phloroglucinols, naphthodianthrones, organosulfur compounds, alkaloids, anthraquinonoids, ginsenosides, pentacyclic triterpene acids, and curcuminoids. The membrane interaction’s applicability to the discovery of phytochemical drug leads is also discussed while referring to previous screening and isolating studies.

## 1. Introduction

Contrary to the general belief that the majority of drugs are synthetic in origin, a number of important medicines are natural products or originate from plants. Representative drugs of plant origin are analgesic morphine (from *Papaver somniferum*), local anesthetic cocaine (from *Erythroxylum coca*), anticholinergic atropine (from *Atropa belladonna*), antimalarial quinine (from *Cinchona officinalis*), and cardiotonic digitoxin (from *Digitalis purpurea*). Plants serve not only as crude drugs, over-the-counter drugs, and nutraceuticals, but also as natural resources for the discovery and development of novel drugs [1]. Herbs and spices have a traditional history of use for health maintenance and disease prevention [2]. The consumption of dietary botanical supplements has also been increasing worldwide because of their relatively low costs and the need for alternative medicines.

Medicinal properties and health benefits of plants are attributed to their bioactive components, phytochemicals. A variety of phytochemicals are known to possess antitumor (antiproliferative, cancer-preventive, and apoptosis-inducing), antimicrobial (microbial growth-inhibiting, antibacterial, and antifungal), anti-inflammatory, analgesic, anesthetic, antioxidant (lipid peroxidation-inhibiting and radical scavenging), neuroprotective, and antiplatelet (platelet aggregation-inhibiting and antithrombotic) activity. These diverse bioactivities would provide plants with disease-preventive and therapeutic potentials. However, detailed knowledge about the mode of action of phytochemicals is still lacking.

The molecular mechanism of phytochemicals has been conventionally interpreted or theorized by the effects on enzymes, receptors, ion channels, transporters, and biological pathways. However, the broad pharmacological spectra of phytochemicals are not necessarily accounted for by only one action on a specific target. Because different phytochemicals are able to modulate the activity of seemingly (structurally and functionally) unrelated proteins, the functionality of plants, which matches with the purpose of medicinal use, is very likely to be produced by a common mechanism of phytochemicals. A ratio of lipids to proteins is approximately one in typical biomembranes. It is requisite for phytochemicals to penetrate into or diffuse through the lipid barriers to reach the site of action. Various events associated with phytochemicals occur in the membrane lipid environments or through the membrane lipid bilayers. Therefore, the interaction with biomembrane-constituting lipids is referred to as one of the important mechanisms underlying the diverse effects of phytochemicals. Phytochemicals are presumed to affect membrane physicochemical properties, including fluidity, microviscosity, order, elasticity, and permeability as well as membrane-acting drugs such as anesthetic, adrenergic, cholinergic, non-steroidal anti-inflammatory, analgesic, antitumor, antiplatelet, antimicrobial, and antioxidant ones. A novel mechanistic point of view of phytochemicals would lead to a better understanding of their pharmacological effects, an insight into their medicinal potentials, and a strategic implication for discovering drug leads from plant resources.

This review is organized as follows. The membrane interactions of conventional and clinically important drugs are presented first as one of their essential molecular mechanisms. Different classes of phytochemicals with membrane interactivity are reviewed next. Experimental applicability of the membrane interaction to the discovery of phytochemical drug leads is also discussed while referring to previous application research. Kopeć *et al.* [3] recently published an excellent review in which they reviewed the molecular dynamics simulations of the interactions between lipid bilayer membranes and various molecules including drugs and plant extracts.

## 2. Membrane-Interactive Drugs

Lipid bilayers not only form a barrier between the cytoplasm and the external environment of cells but also regulate the activity of membrane-embedded or transmembrane proteins by providing them with the localization platform of the membrane domain. Besides membrane-associated receptors, enzymes, and ion channels, membrane lipids are an important acting site for a variety of drugs, such as anesthetic, analgesic, anti-inflammatory, antitumor, antimicrobial, antithrombotic, cardiovascular, and antioxidant ones. These drugs interact with biomembrane-constituting lipids to change the membrane physicochemical properties [4]. The membrane interactions of drugs associated with their pharmacological effects are presented next.

### 2.1. Anesthetics and Anesthesia-Related Drugs

Although the exact molecular mechanism is still an open question in regard to general anesthetics, local anesthetics, and anesthesia-related agents (sedatives, antinociceptives, and anesthetic adjuncts), such drugs have been considered to target or specifically bind to γ-aminobutyric acid type-A (GABA_A_) receptors, *N*-methyl-d-aspartate (NMDA) receptors, or voltage-gated sodium channels as a positive allosteric GABA_A_ receptor modulator or a GABA_A_ receptor agonist like alkylphenol anesthetics, inhalational anesthetics, benzodiazepines, and barbiturates, as an NMDA receptor antagonist like ketamine, and as a sodium channel blocker like lidocaine, bupivacaine, prilocaine, and procaine. However, the controversy as to whether the primary site of action is membrane proteins, membrane lipids or both still remains unresolved. Considering the substantial difference in chemical structure, that a specific binding site within receptors or ion channels is common among all anesthetics seems unlikely. The amphiphilic molecules of anesthetic drugs potentially interact with lipid bilayers to modify membrane organization, dynamics, and physicochemical properties, with the resultant alterations of both biomembrane function and membrane-associated protein conformation, and the subsequent modulation of receptor and ion channel activities, thereby producing diverse effects including intrinsic anesthetic and cardiovascular ones [3,5].

While propofol positively allosterically modulates GABA_A_ receptors, this alkylphenol anesthetic preferentially locates in the hydrophobic deeper regions of lipid bilayers and structure-specifically increases the membrane fluidity or decreases the membrane microviscosity at clinically relevant concentrations [6,7]. Inhalational anesthetic cyclopropane, nitrous oxide, and xenon also act on phospholipid bilayer membranes and synaptic membranes to cause membrane fluidization or disorder [8,9]. Besides specifically binding to GABA_A_ or NMDA receptors, barbiturates (thiopental, pentobarbital, and phenobarbital), benzodiazepines (diazepam and midazolam), and ketamine interact with synaptic and biomimetic membranes to change their physicochemical properties including membrane fluidity [10,11,12]. The hydrophobic interactions between membrane proteins and their surrounding lipids provide an energetic coupling between protein conformation and lipid bilayer elasticity. The activity of GABA_A_ receptors is regulated by the lipid bilayer elasticity that is modified by membrane-interactive anesthetics [13].

Local anesthetics block peripheral nerves at relatively high concentrations and exert either beneficial or adverse effects on hearts at lower concentrations. In the mechanistic protein-interacting theory, local anesthetics in charged form are presumed to bind to the intracellular or cell-interior sites of voltage-gated sodium channels embedded in membrane lipid bilayers for inhibiting the generation and conduction of action potentials, whereas local anesthetic molecules are absolutely required to be in uncharged form for diffusing through the lipid barriers of nerve sheaths and penetrating into the lipid bilayers of neuronal membranes to reach the acting sites. Amphiphilic lidocaine, prilocaine, bupivacaine, ropivacaine, and mepivacaine interact with biomimetic membranes consisting of phospholipids and cholesterol to increase membrane fluidity with potencies correlating to their relative anesthetic effects [14,15]. Lidocaine and dibucaine also increase the elasticity of phospholipid bilayer membranes or decrease membrane thickness [16,17]. Because the gating process of sodium channels depends on the state of membrane lipids surrounding channel proteins, the activities of ion channels are closely related to or modulated by changes in the lipid bilayer property [18]. Sodium currents and action potentials are blocked by chemicals and drugs that increase the fluidity of neuronal and cardiomyocyte membranes [19,20]. The conformation of membrane-embedded proteins is associated with lipid bilayer elasticity to regulate voltage-gated sodium channels [21], and local anesthetics decrease the elasticity of membranes [22].

While general and local anesthetics structure-specifically act on the neural and cardiovascular system, they interact with biomembranes to increase the membrane fluidity in a manner depending on their molecular structures [6,14]. Membrane-interactive pipecoloxylide bupivacaine and ropivacaine induce membrane fluidization, which is discriminated between local anesthetic stereoisomers [15,23]. The relative potencies of these membrane interactions are consistent with those of anesthetic and cardiotoxic effects of anesthetics.

### 2.2. Adrenergic and Cholinergic Drugs

Neurotransmitters modulate the activities of receptors directly by binding to the relevant receptor proteins and indirectly by diffusing into postsynaptic membranes and altering the membrane physicochemical property to shift the receptor conformational equilibrium [24]. As well as such membrane-acting neurotransmitters, adrenergic and cholinergic drugs are able to show neuropharmacological features by the combined interactions with receptor proteins and with membrane lipids.

Beta_2_-adrenoceptor agonists are effective in managing pulmonary diseases due to their bronchodilation activity. The membrane interaction characteristics of ultra-long acting β_2_-agonist indacaterol and less efficient salmeterol reflect the pharmacological difference between them [25].

Beta_1_-adrenoceptor antagonists are perioperatively used to reduce the cardiac disorders associated with general anesthesia. Unlike β_1_-selective blockers (such as esmolol, landiolol, atenolol, and metoprolol), nonselective blockers (such as propranolol, carvedilol, alprenolol, and oxprenolol) interact with neuronal mimetic membranes and lipid raft model membranes to increase their fluidity at clinically relevant concentrations [26,27,28]. The localization in caveolae/lipid rafts is prerequisite to β_2_-adrenoceptors, but not β_1_-adrenoceptors, for physiological signaling. Because membrane fluidization causes the decrease of β_2_-adrenoceptor functions, membrane-fluidizing nonselective β_1_-blockers could reduce the β_2_-adrenoceptor activity by interacting with membrane lipid rafts together with antagonizing β_1_-adrenoceptors by interacting with β_1_-adrenoceptor proteins, thereby producing the nonselective β_1_-blockade. Both selective and nonselective β_1_-blockers are also able to act on mitochondrial mimetic membranes and increase their fluidity. Their induced membrane fluidization independent of blocking β_1_-adrenergic receptors is contributable to the antioxidant cardioprotective effects of β-blockers [28].

Vasodilator carvedilol is a nonselective β-blocker and α_1_-selective blocker that is used for not only treating hypertension and congestive heart failure but also protecting cell membranes from lipid peroxidative damages. Carvedilol and its metabolites increase the fluidity of phospholipid bilayer membranes to exert neuroprotective effects [29].

Cholinergic drugs, both muscarinic agonists and antagonists, interact with rat brain membranes to increase their fluidity [30]. Although muscarinic agonist oxotremorine increases the membrane fluidity of rat lymphocytes, its membrane-fluidizing effect is inhibited by muscarinic antagonist atropine [31].

### 2.3. Non-Steroidal Anti-Inflammatory Drugs and Analgesics

Non-steroidal anti-inflammatory drugs (NSAIDs) possess potent analgesic and antipyretic effects and possibly preventive effects against cancers and Alzheimer’s disease, although their chronic use may produce gastrointestinal injuries. Their therapeutic and pathogenic properties are exclusively attributed to the inhibition of cyclooxygenase (COX), constitutive COX-1 and inducible COX-2, both of which are responsible for the biosynthesis of prostanoids. Since almost all NSAIDs are weakly acidic amphiphilic molecules, they potentially act on membrane lipids. Besides the inhibition of enzymatic activity, NSAIDs cause the interaction with cell membranes to change their fluidity and permeability, accounting for not only COX inhibition-relating effects but COX-independent effects as well [32]. Because COX-1 and COX-2 are integral membrane proteins and their substrates must partition into lipid bilayers, the lipid environments surrounding such proteins modulate COX-catalytic reactions [33]. Therefore, the pharmacological effects of NSAIDs are mechanistically related to their induced alterations of the membrane physicochemical property.

Aspirin, the most common NSAID, increases the fluidity of lipid bilayers [34] and causes the structural and functional perturbation of membranes, including the inhibition of raft formation by locally disrupting the membrane organization [35]. Non-selective COX inhibitors (NSAIDs to inhibit both COX-1 and COX-2) indomethacin, ibuprofen, naproxen, piroxicam, tenoxicam, and diclofenac act on the hydrophobic cores and the surface head groups of artificial phosphatidylcholine membranes and biological membranes [4], with a resultant increase of the membrane fluidity and the subsequent modulation of membrane-associated enzyme activities [36]. In contrast to NSAID-induced membrane fluidization, other studies showed that indomethacin and piroxicam increase the microviscosity (reciprocal value of fluidity) of human neutrophil membranes and phospholipid liposomal membranes together with inhibiting chemoattractant-induced neutrophil aggregation [37] and that aspirin and salicylate decrease the membrane fluidity of human platelets and erythrocytes or increase the lipid order of phosphatidylcholine bilayers [38,39]. Given the opposing changes in membrane fluidity, the membrane effects of NSAIDs may be modified or masked by the difference in membrane compositions, the presence of counter-ions, and/or the reaction between NSAIDs and membrane proteins [40].

Lornoxicam and meloxicam to preferentially inhibit COX-2 have greater ability to change the membrane fluidity of egg yolk phosphatidylcholine unilamellar vesicles than piroxicam and tenoxicam to inhibit both COX-1 and COX-2, correlating to the selectivity of COX inhibition [41]. Licofelone, an inhibitor of both COX and 5-lipoxygenase, decreases the membrane fluidity of HCA-7 colon cancer cells together with inhibiting the epidermal growth factor receptor signaling and inducing the HCA-7 cell apoptosis [42]. Although rat colonocyte membranes are more fluid in the carcinogen-induced colon carcinogenesis, selective COX-2 inhibitors celecoxib and etoricoxib restore such increased membrane fluidity or rigidify the membranes in association with the inhibition of proliferation, migration, and invasion of colon cancer cell lines [43].

Aspirin, ibuprofen, naproxen, indomethacin, and piroxicam interact electrostatically and hydrophobically with the polar head groups and the acyl chains of membrane phospholipids, respectively, resulting in changes of membrane fluidity and permeability [32]. The membrane interactions, independent of COX inhibition, are likely to underlie beneficial (anti-inflammatory, analgesic, and antitumor) effects and adverse (peptic gastrointestinal ulceration and bleeding) effects of NSAIDs [44]. Such a membrane-interacting mechanism is also contributable to COX inhibition through modification of the lipid environments surrounding membrane enzymes with a resultant change of protein conformation and the subsequent inhibition of COXs.

Opioid or narcotic analgesics exert pharmacological effects by binding to opioid receptors (μ-, κ-, and δ-receptor). Among them, morphine selectively acts on μ-receptors and produces significant analgesia that is used to relieve and manage the severe pain effectively. In addition, morphine decreases the membrane fluidity by interacting with rat erythrocyte membranes, not with opioid receptors [45].

Ketamine is an NMDA receptor antagonist that is clinically used for the purpose of anesthesia induction and analgesia. In addition to antagonizing at NMDA receptors, it interacts with synaptic and artificial membranes to change their properties, which is responsible for its anesthetic effect [12].

Alkylphenol thymol and eugenol are useful for dental pulp sedation and analgesia in dentistry. They show neuroactivity not only by the allosteric positive modulation of GABA_A_ receptors [46] but also by the interaction with neuronal membranes to increase their fluidity [47].

### 2.4. Antitumor and Antiproliferative Drugs

Cellular membranes and membranous organelles are referred to as one of crucial targets for antitumor, anticancer, or cancer chemopreventive agents [48]. The activation and suppression of cell proliferation occur in the membrane lipid environments, which are governed by the physicochemical properties of lipid bilayers [49]. The proliferative ability of tumor cells is closely linked to the membrane fluidity. Because the membranes of neoplastic and metastatic cells are more fluid than their normal counterparts due to the reduced cholesterol composition and the elevated unsaturated phospholipid composition, membrane-rigidifying drugs would counteract the increased membrane fluidity of tumor cells. Many antitumor drugs affect tumor cell proliferation by inhibiting tumorigenesis-related enzymes, inducing apoptosis, modulating proliferative signal transduction, arresting cell cycle progression, and altering receptor functions or by a combination of these modes of action. While COX-2 plays an important role in tumorigenesis, the modification of membrane fluidity leads to COX inhibition [50]. The altered fluidity disturbs the membrane lipid environments optimal for the protein conformation of tumorigenesis-relevant enzymes and receptors. Apoptosis is inducible through the membrane fluidity alteration of tumor cells [51]. The cell cycle is accompanied by the fluidity change in cell membrane dynamics, and the membranes of resting cells are less fluid than those of proliferating ones [49].

Antitumor drugs like cisplatin and doxorubicin promote apoptotic cell death. They modulate the fluidity and lipid composition of cellular membranes together with altering the signal transduction of cell proliferation [52]. DNA-damaging cisplatin induces apoptosis through the disruption of plasma membranes, although unlike the other antitumor drugs, it increases the membrane fluidity of human colon cancer cells [53]. Antineoplastic antibiotic doxorubicin decreases the membrane fluidity of the human leukemia cell line [54] or increases the membrane microviscosity of erythrocytes from acute myeloid leukemia patients [55]. Although its primary mode of action is to inhibit an estrogen receptor, tamoxifen is also able to decrease the membrane fluidity of human breast cancer cells and modulate various signaling pathways, inducing apoptotic cell death [56]. Antineoplastic drugs derived from arylchloroethylurea rigidify biomimetic membranes with the potency depending on membrane lipid components [57]. Alkylating drugs chlorambucil, nitrogen mustard, and trenimon decrease the membrane fluidity of Ehrlich ascites tumor cells, but not that of the cells resistant to nitrogen mustard [58]. Mitotic inhibitor paclitaxel rigidifies unsaturated phospholipid bilayer membranes by acting on the hydrophobic core of unilamellar vesicles [59].

### 2.5. Antimicrobial Drugs and Antibiotics

Antimicrobial drugs interact with bacterial and fungal cell membranes, resulting in changes of membrane fluidity and permeability. Such membrane interactions, at least in part, mechanistically underlie the effects of drugs, including antibiotics, to affect or inhibit the growth of bacteria and fungi.

Disinfectant chlorhexidine and surface-acting benzalkonium chloride interact with phospholipid liposomal membranes and bacterial cell membranes to decrease their fluidity [60]. A quaternary ammonium disinfectant also acts on liposomes prepared with phospholipids from *Staphylococcus aureus* and increases the membrane fluidity [61].

While fluoroquinolone antibiotics primarily inhibit intracellular DNA gyrase and topoisomerase IV, they must enter bacterial cells through the membrane lipid bilayers to affect the activities of these enzymes. Ciprofloxacin, one of such antibiotics, interacts preferentially with anionic phospholipids to change the fluidity and order of lipid bilayer membranes [62]. Aminoglycoside antibiotics streptomycin and isepamicin are able to interact with negatively charged membranes and increase their permeability [63]. Macrolide antibiotic azithromycin interacts with phospholipid bilayers at the hydrophilic/hydrophobic interface to increase membrane fluidity and affect membrane organization and permeability [64]. Azithromycin also modifies the fluidity of liposomal membranes and mouse macrophage membranes [65]. Polyene antibiotic amphotericin B and imidazole antifungal miconazole specifically act on ergosterol-containing fungal cell membranes to alter membrane permeability, and both drugs also decrease the membrane fluidity of human polymorphonuclear leukocytes at antimicrobial-relevant concentrations [66]. Non-polyene antibiotic primycin with a broad antimicrobial spectrum has the property to interact with the plasma membranes of *Candida albicans* and rigidify them [67].

### 2.6. Antioxidant and Lipid Peroxidation-Inhibitory Drugs

A wide range of diseases are pathogenetically related to oxidative stress, an imbalance between oxidant production and antioxidant defense in the biological system. Surgery and anesthesia enhance the generation of oxygen-derived reactive species and weaken the *in vivo* defense against their attacks. Oxidative stress is also implicated in ischemia-reperfusion and cardiovascular injuries. Because reactive oxygen species damage biomolecules by peroxidizing membrane lipids, antioxidant drugs to inhibit lipid peroxidation are expected to protect against many pathological states associated with the oxidative stress.

The susceptibility to reactive oxygen species depends on the fluidity of lipid membranes [68]. While thiopental, diazepam, midazolam, ketamine and their related drugs show the inhibitory effects on lipid peroxidation in addition to their intrinsic anesthetic activity, all of them interact with biological and artificial membranes to increase membrane fluidity [69]. Perioperatively used β_1_-blockers inhibit lipid peroxidation simultaneously with fluidizing the membranes [28]. The propagation of both oxidant and antioxidant molecules in lipid bilayers is a rate-limiting step of lipid peroxidation [70]. Membrane fluidization is preferable for producing the antioxidant effects because antioxidant drug molecules can interact with radicals more efficiently in fluid membranes [71]. The membrane interaction to increase the fluidity would contribute to the inhibition of membrane lipid peroxidation cooperatively with the radical scavenging.

On the other hand, certain antioxidants decrease the fluidity of membranes. Such membrane-acting drugs partition into the internal hydrophobic regions and affect the motional freedom of molecules in lipid bilayers. Their induced membrane rigidification is presumed to hamper the diffusion of radicals and decrease the kinetics of radical reactions in the membrane lipid environments, resulting in the inhibition of lipid peroxidation [72].

Taken together, the modification (increase or decrease) of membrane fluidity is mechanistically responsible for the antioxidant effects of drugs.

### 2.7. Antiplatelet and Platelet Aggregation-Inhibitory Drugs

Antiplatelet or antithrombotic drugs to inhibit platelet aggregation interfere with different platelet stimulants such as collagen, epinephrine, thrombin, adenosine 5′-diphosphate (ADP), and platelet-activating factor (PAF). Besides individual receptors of these agonists, the platelet membrane is a common site of action for drugs with antiaggregatory activity [73]. Membrane fluidity is one of the important factors to regulate the function and structure of platelets. Since a change in membrane fluidity is linked to the aggregability of platelets [74], almost all antiplatelet drugs affect the membrane fluidity of platelets, with a resultant modification of the activity of phospholipase C and the subsequent inhibition of phosphoinositide breakdown, thereby altering intracellular Ca^2+^ mobilization, resulting in the inhibition of platelet aggregation [75].

Membrane-rigidifying doxorubicin inhibits collagen- and ADP-induced platelet aggregation [76]. Midazolam and ketamine, which inhibit collagen- and thrombin-induced platelet aggregation, directly act on platelet membranes to change their fluidity [77,78].

The modification of platelet membrane fluidity mechanistically participates in the antiplatelet effects of drugs cooperatively with the action on platelet aggregation-relevant receptors and enzymes.

## 3. Phytochemicals with Membrane Interactivity

As described in Section 2, membrane interactions are essentially or at least partly responsible for the pharmacological effects of various drugs. If phytochemicals similarly show the membrane interactions associated with disease-preventive and therapeutic potentials, they would be the candidates for drug leads, which may lead to the development of novel drugs and enhance the medicinal values of plants containing them.

Different techniques such as differential scanning calorimetry (DSC), electron spin resonance (ESR), nuclear magnetic resonance (NMR), electron paramagnetic resonance (EPR), X-ray diffraction, and fluorometry are used for studying the membrane interactions of phytochemicals. Of these methods, fluorescence polarization (FP) or fluorescence anisotropy (FA) have been most frequently applied to plant preparations, isolated phytochemicals, and phytochemical synthetics to analyze their induced changes in membrane fluidity. The term “membrane fluidity” may mean a combination of different kinds of membrane component mobility such as the flexibility of membrane phospholipid acyl chains, the lateral or transverse diffusion of molecules in lipid bilayers, and the membrane lipid phase transition. Monochromatic light passes through a vertical polarizing filter and excites naturally fluorescent molecules or fluorescent probe-labeled molecules. The molecules oriented properly in the vertically polarized plane absorb the light, become excited, and emit the light (fluorescence) which is measured in the horizontal and vertical planes. Both polarization and anisotropy are derived from the measured vertical and horizontal fluorescence intensities, and their calculated values are mathematically related and interconvertible.

As well as biological membranes, biomimetic membranes prepared with authentic or extracted lipids have been widely used to evaluate the membrane interactions of phytochemicals. The advantages of using such protein-free lipid membranes are that one can easily manipulate the reaction condition and the membrane lipid composition, focus on the interaction with membrane lipids, avoid the interference from other membrane components, and determine the membrane responses to phytochemicals quantitatively. Membrane specimens are prepared to mimic the lipid compositions of cellular and plasma membranes of interest. The most frequently used biomimetic membrane is the liposome or unilamellar vesicle with concentric lipid layers in which an aqueous volume is enclosed by membranous lipids. A unilamellar vesicle is characterized by a single lipid bilayer consisting of inner and outer leaflets. Glycerophospholipid, the major phospholipid component in biological membranes, consists of a glycerol backbone on which two fatty acids are usually esterified at the position of *sn*-1 and *sn*-2. Numbering with the prefix “*sn*” characterizes the carbon atoms of glycerol stereospecifically. The third carbon atom of a glycerol backbone supports the polar head group composed of choline, ethanolamine, serine, inositol, and glycerol, which are linked to a negatively charged phosphate group. Representative phospholipids used for preparing biomimetic membranes are 1,2-dipalmitoyl-*sn*-glycero-3-phosphocholine (DPPC), 1,2-dioleoyl-*sn*-glycero-3-phosphocholine (DOPC), 1,2-dimyristoyl-*sn*-glycero-3-phosphocholine (DMPC), 1-palmitoyl-2-oleoyl-*sn*-glycero-3-phosphocholine (POPC), 1-stearoyl-2-linoleoyl-*sn*-glycero-3-phosphocholine (SLPC), 1,2-dipalmitoyl-*sn*-glycero-3-phosphoethanolamine (DPPE), 1-palmitoyl-2-oleoyl-*sn*-glycero-3-phosphoethanolamine (POPE), 1-palmitoyl-2-oleoyl-*sn*-glycero-3-[phospho-L-serine] (POPS), 1-stearoyl-2-oleoyl-*sn*-glycero-3-[phospho-L-serine] (SOPS), 1,2-dipalmitoyl-*sn*-glycero-3-phosphoglycerol (DPPG), 1,2-dimyristoyl-*sn*-glycero-3-phosphoglycerol (DMPG), sphingomyelin, and cardiolipin. Cholesterol is also used because it is the most abundant steroid in biological membranes. This lipid comprises four fused cycles in the *trans*-configuration, a hydroxyl group at the 3-position, a double bond between the carbon 5 and 6, and an *iso*-octyl lateral chain at the 17-position.

A variety of fluorophores have been used as fluorescent probes to measure FP and FA of the membranes treated with phytochemicals. They include 1,6-diphenyl-1,3,5-hexatriene (DPH), 1-(4-trimethylammoniumphenyl)-6-phenyl-1,3,5-hexatriene (TMA-DPH), *N*-phenyl-1-naphthylamine (PNA), 1-anilinonaphthalene-8-sulfonic acid (ANS), 2-(9-anthroyloxy)stearic acid (2-AS), 6-(9-anthroyloxy)stearic acid (6-AS), 9-(9-anthroyloxy)stearic acid (9-AS), 12-(9-anthroyloxy)stearic acid (12-AS), and 16-(9-anthroyloxy)palmitic acid (16-AP). These probes penetrate into lipid bilayers to locate in different membrane regions based on their chemical structures and properties, indicating the fluidity change of the membrane region specific to each individual probe. They are subject to the rotational restriction imparted by membrane rigidity or order. When phytochemicals interact with lipid bilayers to rigidify or order the membranes, the produced more rigid or ordered membranes disturb the probe rotation to emit the absorbed light in all directions, resulting in an increase of FP. On the contrary, the membranes fluidized or disordered by phytochemicals facilitate the probe rotation, resulting in a decrease of FP. Compared with controls, increased and decreased FP values mean a membrane fluidity decrease (membrane rigidification) and membrane fluidity increase (membrane fluidization), respectively.

Different classes of membrane-interactive phytochemicals are reviewed next in association with their pharmacological effects.

### 3.1. Flavonoids

**Figure 1:**
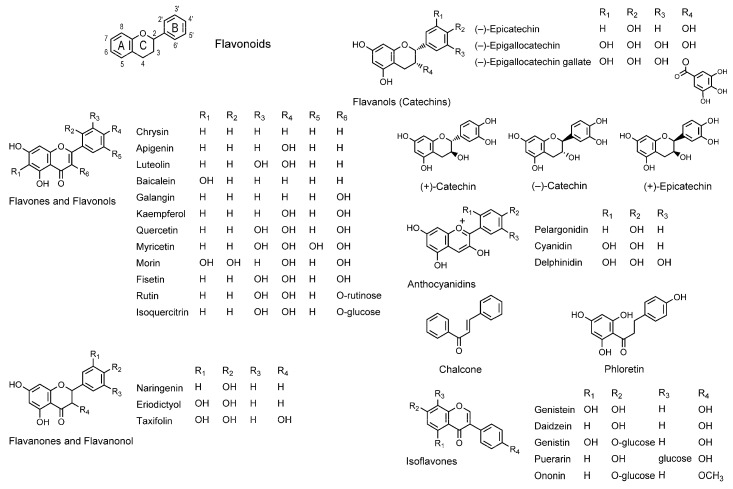
Structures of membrane-interactive flavonoids.

Flavonoids constitute one of the most ubiquitous groups of phytochemicals contained in medicinal plants, nutraceutical plant foods, dietary botanical supplements, and herbal remedies. They have the common skeleton of a chromane ring with aromatic rings attached at the 2- or 3-position that are labeled with A, B, and C (Figure 1). Based on the difference of substituents and the oxidation status of a heterocyclic C ring, flavonoids are divided into flavone, flavonol, flavanone, flavanonol, flavanol (catechin), anthocyanidin with an oxonium ion O^+^ in the C ring, chalcone lacking the C ring, and isoflavone with the C ring at the 3-position. Isoflavones should probably be classified as isoflavonoids, not flavonoids.

Flavonols such as quercetin, kaempferol, rutin (quercetin 3-*O*-rutinoside), and quercitrin (quercetin 3-*O*-rhamnoside) are present in St. John’s wort, the leaves and flowering tops of *Hypericum perforatum* which have been used for mild to moderate depression. Quercetin and myricetin are found in the leaves of ginkgo (*Ginkgo biloba*) which have been used for memory and concentration enhancement, blood flow improvement, and blood clotting inhibition. Both St. John’s wort and ginkgo also contain the flavones apigenin and luteolin. The primary bioactive components in green tea, the product of the plant *Camellia sinensis*, are flavanols such as (–)-epigallocatechin-3-gallate (EGCG) and (–)-epigallocatechin (EGC). Anthocyanidins cyanidin and delphinidin; flavone luteolin; flavonols quercetin, myricetin, and rutin; and flavanols EGC and EGCG are present in *Vaccinium* berries cranberry, bilberry, and blueberry. Dihydrochalcone phloretin is one of the major components in the leaves of apples (wild apple *Malus sieversii* and domesticated apple *Malus domestica*).

Although flavonoids cover very broad pharmacological spectra, the mode of action has been only partially understood. Besides interacting with functional proteins (enzymes, receptors, and ion channels) as the primary target, bioactive flavonoids have been presumed to act on lipid bilayers and modify the membrane physicochemical properties. Arora *et al.* [72] demonstrated that flavonoids (naringenin and rutin) and isoflavonoids (genistein) partition into the hydrophobic cores of SLPC unilamellar vesicles and decrease the membrane fluidity by measuring FA with 6-AS, 12-AS, and 16-AP. Tsuchiya [79] determined the comparative membrane interactions of 22 flavonoids by measuring FP with 2-AS, 6-AS, 9-AS, 12-AS, and 16-AP. His results indicated that flavones (apigenin and luteolin), flavonols (kaempferol, quercetin, and myricetin), flavanones (naringenin and eriodictyol), flavanonols (taxifolin), anthocyanidins (pelargonidin, cyanidin, and delphinidin), flavanols ((–)-epicatechin, EGC, and EGCG), isoflavones (genistein), and chalcones (phloretin) act at 1–10 µM on the deeper regions of liposomal membranes consisting of POPC, POPE, POPS, and cholesterol to decrease the membrane fluidity, which is associated with the inhibition of tumor cell proliferation. Based on 12-AS polarization increases at each 10 µM, cyanidin, quercetin, and EGCG are the most potent in membrane interaction, followed by kaempferol, pelargonidin, phloretin, luteolin, apigenin, myricetin, and genistein. Wu *et al.* [80] treated DPPC and DPPG unilamellar vesicles with flavonoids and isoflavonoids. By measuring FP with DPH, they showed that flavonoids decrease the membrane fluidity with the potency being kaempferol > chrysin > baicalein > quercetin > luteolin, whereas isoflavonoids increase the membrane fluidity with the potency being puerarin > ononin > daidzein > genistin. However, Ajdzanović *et al.* [81] reported the EPR spectroscopic study indicating different membrane interactions of isoflavonoids, where genistein decreases the fluidity of erythrocyte membranes near the hydrophilic surfaces, though daidzein increases that at the membrane deeper regions. Tsuchiya [82] compared the interactions of 17 structurally different flavonoids with biomimetic membranes consisting of POPC, POPE, SOPS, and cholesterol by measuring FP with 2-AS, 6-AS, 9-AS, 12-AS, and 16-AP together with determining their antiproliferative effects. Based on 12-AS polarization increases at each 10 µM, the relative membrane-interacting potency was galangin > quercetin > kaempferol > chrysin > baicalein > fisetin > luteolin > apigenin > myricetin > morin. The comparison of membrane interactivity suggested that 3-hydroxylation of the C ring, non-modification or 3′,4′-dihydroxylation of the B ring, and 5,7-dihydroxylation of the A ring produce the greatest membrane interaction to rigidify membranes. The comparison between aglycone (quercetin) and glycosides (rutin and isoquercitrin, quercetin 3-*O*-glucoside) also indicated that glycosylation significantly reduces the membrane interactivity of flavonoids. Such structural requirement-meeting galangin and quercetin inhibited the proliferation of tumor cells simultaneously with decreasing the fluidity of their membranes. Margina *et al.* [83] treated human U937 monocytes and Jurkat T lymphoblasts with quercetin and EGCG, and then measured FA with TMA-DPH. They found that quercetin and EGCG decrease the membrane fluidity at 10–50 μM, correlating to their inhibitory effects on membrane lipid peroxidation. Membrane-interactive flavonoids are efficiently incorporated into human erythrocytes to protect the membranes against oxidative damage [84] and the bioactivities of flavonoids correlate to their membrane localization and their induced changes in membrane fluidity [85]. Tsuchiya [86] comparatively studied the interactions of eight flavanols with DPPC or DOPC liposomal membranes by measuring FP with PNA and ANS. He revealed that all the tested catechins decrease the membrane fluidity in both hydrophilic and hydrophobic regions of lipid bilayers at micromolar concentrations and that EGCG is the most effective to prevent the membrane-fluidizing effects of hepatotoxic chloroform at 2.5 μM. The following study showed that flavanol stereoisomers interact with biomimetic membranes consisting of phospholipid and cholesterol with the potency being (–)-epicatechin > (+)-epicatechin > (–)-catechin > (+)-catechin [87]. Such stereospecific membrane interactions are likely to be mediated by the different hydrophobicity of geometrical isomers [88] and the chirality of membrane lipid components to discriminate optical isomers [5].

The membrane-interacting properties of flavonoids to modify fluidity, order, and permeability of artificial and cellular membranes are correlated with their antioxidant, antitumor, antiplatelet, antimicrobial, and anti-inflammatory effects as shown by many studies [79,80,82,83,89].

### 3.2. Terpenoids

Terpenoids (or isoprenoids) are widely distributed in herbs and dietary botanical supplements, especially in the essential oils from aromatic plants. The aromatic quality of terpenoids is responsible for the flavor of cinnamon, clove, and ginger, the scent of eucalyptus, and the color of tomato. In addition, the plants containing terpenoids have been traditionally used as herbal medicines. Terpenoids derived from five-carbon (C5) isoprene units are classified by the number of C5 unit into monoterpenoid with 2 × C5 unit, sesquiterpenoid with 3 × C5 unit, diterpenoid with 4 × C5 unit, and triterpenoid with 6 × C5 unit.

**Figure 2:**
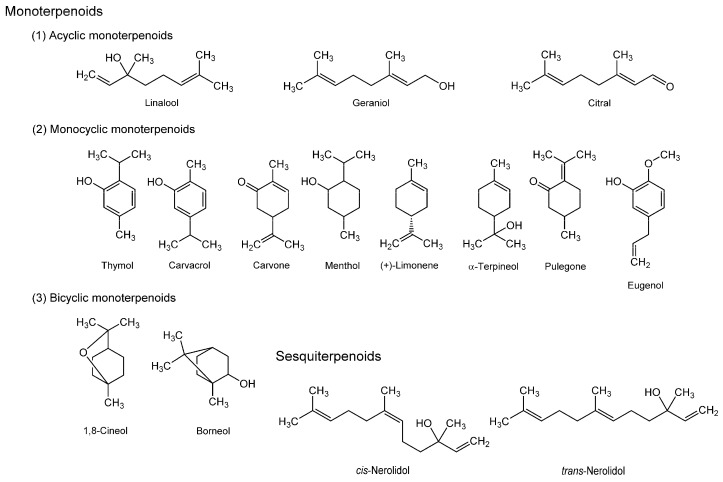
Structures of membrane-interactive terpenoids.

Monoterpenoids in essential oils consist of acyclic, monocyclic, and bicyclic ones (Figure 2). Acyclic monoterpenoids include (1) linalool from coriander (*Coriandrum sativum*) used in cooking and medicinally for diabetes and inflammatory diseases; (2) geraniol contained in rose and palmarosa oil with antimicrobial activity; and (3) citral present in the essential oils from lemon myrtle, lemongrass, lime, lemon, and orange, which are used as a flavoring agent with antimicrobial quality. Monocyclic monoterpenoids include (1) thymol contained at a significantly high concentration in thyme (*Thymus vulgaris*), a spicy culinary herb to be used for antiseptic mouthwash, cough, and bronchitis, and contained in oregano (*Origanum vulgare*), a common culinary herb to be used for stomach, respiratory, and infectious diseases; (2) carvacrol from oregano (*Origanum vulgare* and *Origanum majorana*); (3) carvone contained in caraway (*Carum carvi*) to be used for upset stomach, nausea, and gastrointestinal spasm, and contained in spearmint (*Mentha spicata*) to be used for digestive disorder, fever, and headache; (4) menthol contained in spearmint and peppermint (*Mentha piperita*) to be used for heartburn, nausea, abdominal pain, and gastrointestinal spasm; (5) (+)-limonene, a main odor component in citrus (the family Rutaceae) to be used as a flavoring agent with insecticide activity; (6) α-terpineol contained in the essential oils from laurel (*Laurus nobilis*), rosemary (*Rosmarinus officinalis*), and anise (*Pimpinella anisum*), which are used as an ingredient in perfumes, cosmetics, and flavors; and (7) pulegone contained in horse mint (*Mentha longifolia*), peppermint (*Mentha piperita*), and catnip (*Nepeta cataria*). Thymol and carvacrol may be also classified as terpenoid phenols or phenylpropanoids together with monoterpenoid eugenol, a bioactive component in aromatic plants. The best natural source of eugenol is clove (*Eugenia aromatica* or *Syzygium aromaticum*) that has been used for toothache, upset stomach, nausea, and diarrhea, and for mouth and throat inflammation. Eugenol is also present in oregano (*Origanum vulgare*) and cinnamon (*Cinnamomum verum*) to be used as a spice with potential health benefits. Bicyclic monoterpenoinds include (1) 1,8-cineol (or eucalyptol) contained in the oil from *Eucalyptus globulus*, which is used as an ingredient in mouthwash and cough suppressant and as an insecticide; and (2) borneol contained in cardamom (*Elettaria cardamomum*), nutmeg (*Myristica fragrans*), and ginger (*Zingiber officinale*), all of which are consumed as spices. Ginger is also used for nausea, cold, headache, rheumatism, cardiovascular diseases, sedation, and anticonvulsion in traditional medicine, and for enhancing the penetration of other drugs in modern clinical application.

Sesquiterpenoids include (1) nerolidol contained in the essential oils from bitter orange, ginger, jasmine, lavender, and lemongrass, which have been used as a flavoring agent and a skin penetration enhancer of transdermal drugs; (2) bilobalide contained in ginkgo (*Ginkgo biloba*) to be used for conditions associated with memory disorders like dementia and for eye disorders like glaucoma; (3) valerenic acid contained in valerian, the name of an herb or supplement prepared from *Valeriana officinalis* to be used as a sedative and anticonvulsant for insomnia and migraine treatment.

Representative diterpenoids are (1) miltirone contained in red sage (*Salvia miltiorrhiza*) to be used for renal failure and coronary heart diseases; and (2) ginkgolide A, B, C, J, and M from ginkgo (*Ginkgo biloba*).

Triterpenoid glycosides include ginsenosides Rb_1_, Rb_2_, Rc, Re, Rf, Rg_1_, Rg_2_, Rg_3_, Rh_1_, and Rh_2_ from ginseng that has been traditionally used to improve mood, vitality, and sexual function and for depression, anxiety, immune-stimulation, diabetes, memory problems, and bleeding disorders. The membrane interactions of ginsenosides are reviewed in Section 3.9.

Bard *et al.* [90] found that acyclic monoterpenoid geraniol enhances the permeability of *Candida albicans* whole cells and also increases the fluidity of both *Candida albicans* membranes and DPPC liposomal membranes by DSC and FP measurements with DPH, suggesting the relation between the antifungal activity and membrane interactivity of geraniol. Nowotarska *et al.* [91] showed that monocyclic monoterpenoid carvacrol increases the fluidity of bacterial phospholipid membranes consisting of DPPE, DPPG, and cardiolipin as well as geraniol. Reiner *et al.* [92] reported that thymol, carvacrol, and eugenol not only positively modulate the functions of GABA_A_ receptors but also modify the properties of lipid membranes. Their FA experiments with DPH and TMA-DPH indicated that these terpenoids act on DPPC multilamellar vesicles to increase the membrane fluidity at 50–200 µM [93]. Mendanha *et al.* [94] studied the membrane effects of various terpenoids on mouse fibroblasts and human erythrocytes by EPR spectroscopy with a spin label 5-doxyl stearic acid. They revealed that all the tested terpenoids (nerolidol, menthol, pulegone, carvone, (+)-limonene, α-terpineol, and 1,8-cineol) increase the fluidity of both cell membranes and exert cytotoxic effects on fibroblast cells, with sesquiterpenoid nerolidol being more potent than monoterpenoids. While acyclic and monocyclic monoterpenoids show antibacterial activity, Zengin and Baysal [95] demonstrated that linalool, 1,8-cineol, and α-terpineol alter the permeability of outer membranes and the function of cell membranes of *Staphylococcus aureus* and *Escherichia coli* at bacterial growth-inhibitory concentrations by a scanning electron microscopic study. Although Nomura and Kurihara [96] used soybean phospholipid liposomes as an olfactory cell model for terpenoids and odorants, they revealed that acyclic monoterpenoid citral decreases the membrane fluidity at high micromolar concentrations but other odorants increase the membrane fluidity by measuring FP with DPH. Yin *et al.* [97] investigated the interaction of bicyclic monoterpenoid borneol with DPPC bilayer membranes and found its property to increase membrane fluidity. Ginkgo contains diterpenoids and sesquiterpenoids. When mice were orally administered the extract of *Ginkgo biloba* leaves (100 mg/kg/day) for three weeks, the fluidity of neuronal membranes was increased while also improving the short-term memory in a passive avoidance paradigm in the *in vivo* study of DeFeudis and Drieu [98].

The membrane interactions of terpenoids to modify membrane property and organization have been implicated in their antifungal, antibacterial, anesthesia-potentiating, neuro-protective, antioxidant, and antiparasitic effects [91,93,99,100].

### 3.3. Stilbenoids

**Figure 3:**
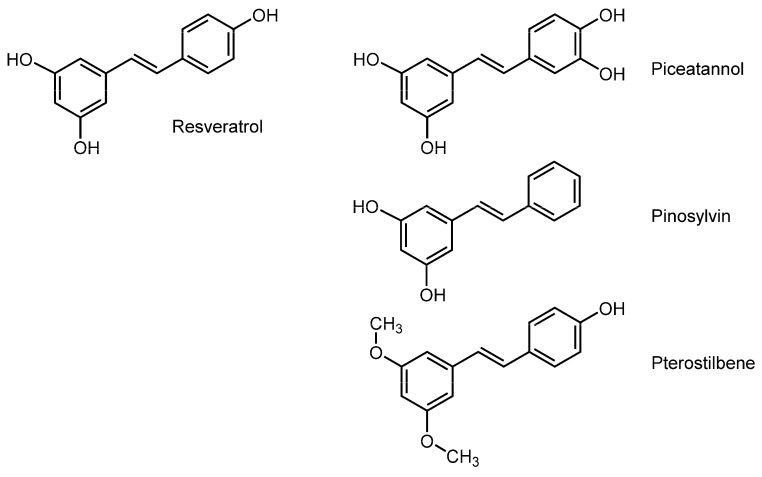
Structures of membrane-interactive stilbenoids.

Stilbenoids are polyphenolic stilbenes, including resveratrol (*trans*-3,5,4′-trihydroxystilbene), piceatannol (*trans*-3,3′,4,5′-tetrahydroxystilbene), pterostilbene (*trans*-3,5-dimethoxy-4′-hydroxystilbene), pinosylvin (*trans*-3,5-dihydroxystilbene) and their derivatives (Figure 3). Many of them have the characteristics of phytoalexin, a secondary metabolite that is synthesized by the infectious stimuli and accumulated at the area of pathogen infection to exhibit antimicrobial activity. Japanese knotweed (*Fallopia japonica*) is a concentrated source of resveratrol. Resveratrol and pterostilbene are contained in grapes at a high concentration and in other berry fruits such as blueberry, cranberry, and bilberry. Resveratrol is also present in peanuts, especially sprouted peanuts. Stilbenoids have been expected to exert antioxidant, antitumor, anti-inflammatory, anti-arteriosclerotic, and dementia-preventive effects.

Tsuchiya *et al.* [101] compared the interactions of resveratrol with tumor cell mimetic and normal cell mimetic membranes, both of which consist of DPPC, POPC, and cholesterol but with a different unsaturation degree of phospholipids and a different composition of cholesterol, by measuring FP with DPH and TMA-DPH. They found that resveratrol decreases the fluidity of tumor cell membranes (phosphatidylcholine acyl chain 18:1/16:0 ratio = 1.0 and 20 mol % cholesterol) at micromolar concentrations much more effectively than that of normal cell membranes (18:1/16:0 ratio = 0.5 and 40 mol % cholesterol) by acting on the hydrocarbon cores of lipid bilayers. Selvaraj *et al.* [102] also reported the NMR spectroscopic study in which resveratrol rigidifies liposomal membranes by preferentially acting on the polar head groups with the potency correlating to its cytotoxic effect on a breast cancer cell line. In contrast, Brittes *et al.* [103] reported that resveratrol increases the membrane fluidity of DPPC unilamellar vesicles by penetrating into the acyl chain regions but positioning its hydroxyl groups near the phospholipid head group regions through fluorescence quenching and FA experiments with 6-AS, 9-AS, ANS, and TMA-DPH. Wesołowska *et al.* [104] showed that stilbenoids structure-dependently interact with the head group regions of DPPC and DMPC liposomal membranes to affect the phospholipid phase transition with the potency being piceatannol > resveratrol by DSC, fluorescence, and ESR spectroscopic studies.

The membrane interactions of stilbenoids are mechanistically related to their antitumor, antibacterial, and antioxidant effects as supported by many studies [101,102,103,104,105].

### 3.4. Capsaicinoids

**Figure 4:**
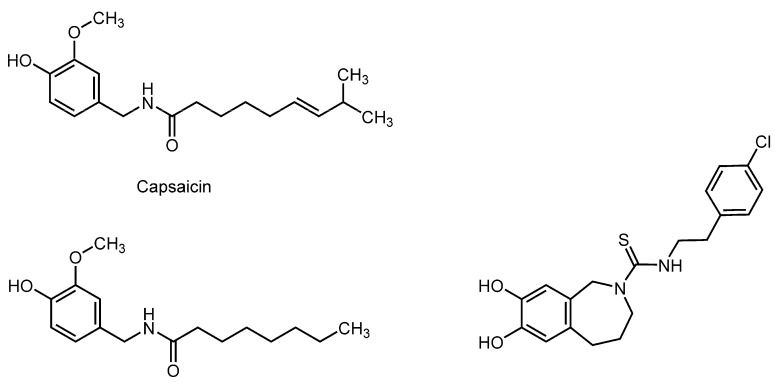
Structures of membrane-interactive capsaicinoids.

Capsaicin (8-methyl-*N*-vanillyl-*trans*-6-nonenamide), a pungent component in chili pepper belonging to the genus *Capsicum*, is one of the bioactive capsaicinoids (Figure 4). Its structurally related dihydrocapsaicin, nordihydrocapsaicin, homodihydrocapsaicin, and homocapsaicin are also included in capsaicinoids. On the initial application, capsaicin causes the excitation of peripheral sensory nerve endings to depolarize and generate action potentials, resulting in burning pain and hyperalgesia. With repeated or prolonged application, however, the stimulation with capsaicin is followed by desensitization and subsequent conduction block and nociceptor inhibition, thereby producing analgesia. Capsaicin has been used as an analgesic in topical ointment, spray, dermal patches, and cream, and is currently used for the treatment of painful conditions like diabetic neuropathy and rheumatoid arthritis.

Meddings *et al.* [106] showed that capsaicin increases the fluidity of non-neuronal plasma membranes at micromolar concentrations by FP experiments with DPH and TMA-DPH. Tsuchiya [107] also studied the interactions of capsaicin and its structural analog *N*-vanillylnonanamide with liposomal membranes consisting of DPPC, POPC, or a mixture of POPC and cholesterol by measuring their induced FP changes with PNA, DPH, and TMA-DPH. Both capsaicinoids, especially capsaicin, were found to show concentration-dependent biphasic effects to fluidize platelet and bacterial cell mimetic membranes at a relatively low concentration (~50 µM) but rigidify at relatively high concentrations (100–500 µM). Several spices including chili pepper, turmeric, and garlic have hypolipidemic activity. Since hyperlipidemic conditions should affect membrane fluidity, Kempaiah and Srinivasan [108] investigated the effects of capsaicin on hypercholesterolemic rats by maintaining them on diets containing 0.015% capsaicin for eight weeks, and then determining the membrane fluidity of erythrocytes by ESR spectroscopy and FA measurement with DPH. Consequently, they found that capsaicin increases the membrane fluidity and reverses the decreased membrane fluidity of hypercholesterolemic rats. The membrane-interacting property of capsaicin was also confirmed by another *in vivo* study by Prakash and Srinivasan [109], in which rats were maintained on diets containing 3.0% chili pepper or 0.01% capsaicin for eight weeks and the fluidity changes of intestinal brush-border membranes were determined by measuring FP with DPH. Their results indicated that both chili pepper and capsaicin increase the membrane fluidity in ileum and jejunum together with stimulating the activities of different enzymes in intestinal mucosa, suggesting an increased absorptive surface of the small intestine through the capsaicin-induced alteration in membrane lipid dynamics.

Capsaicin activates a transient receptor potential vanilloid type-1 (TRPV1) receptor to exert analgesic or algesic effects depending on its concentration. In addition, Lundbæk *et al.* [21] reported that capsaicin and its antagonist capsazepine cause the functional modification of various membrane proteins at micromolar concentrations by inducing the alteration of lipid bilayer elasticity that is sufficient to change the conformation of membrane-embedded proteins. They also showed that this membrane interaction mechanism is responsible for the regulation of voltage-dependent sodium channels by ~100 µM capsaicin.

The TRPV1 receptor is a nonselective cation channel, which plays an important role in modulating nociceptive and pain transmissions in the peripheral and central nervous system. Binshtok *et al.* [110] suggested that capsaicin could transport the sodium channel blocker *N*-(2,6-dimethylphenylcarbamoylmethyl)triethylammonium bromide (QX-314) to nociceptors by the TRPV1-mediated entry, producing nociceptive-selective analgesia. Externally applied QX-314 has no effects on sodium channels because it is unable to pass the lipid bilayers of cellular membranes due to its permanently charged property, but it can gain an access to the cell interiors through TRPV1 channels opened by capsaicin, resulting in the block of sodium channels. This transmembrane access route occurring only on nociceptors is usable by lidocaine and bupivacaine as well as QX-314. Therefore, the combination of local anesthetics with capsaicin would lead to the ideal analgesia-restricted local anesthesia that preserves motor and autonomic responses. Capsaicin, which can fluidize neuronal membranes at low micromolar concentrations, may cooperatively potentiate the membrane-fluidizing effects of lidocaine and bupivacaine [111].

The analgesic, antinociceptive, local anesthesia-potentiating, and hypolipidemic potentials of capsaicinoids have been mechanistically interpreted by membrane interactions [21,108,111].

### 3.5. Phloroglucinols and Naphthodianthrones

**Figure 5:**
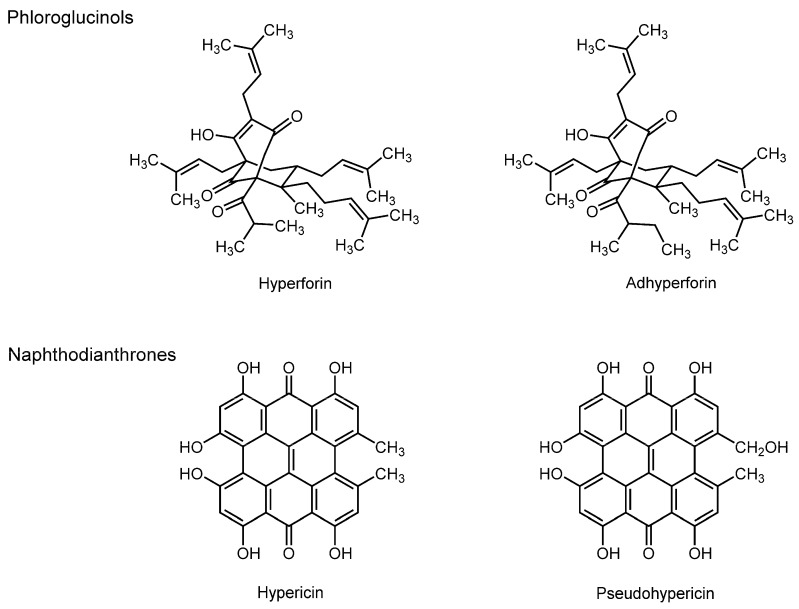
Structures of membrane-interactive phloroglucinols and naphthodianthrones.

The popular plant name “St. John’s wort” corresponds to several species of the genus *Hypericum* (*Hypericum perforatum*, *Hypericum hirsutum*, *Hypericum patulum*, and *Hypericum olympicum*), which have been traditionally used for mild to moderate depression and also as a topical remedy for infected wounds and inflammatory skin diseases [112]. Besides flavonoids (such as rutin, quercetin, quercitrin, and isoquercitrin), two classes of phytochemicals, prenylated phloroglucinols and naphthodianthrones, are isolated from the stems, leaves, and flowers of *Hypericum perforatum* as bioactive components. The former includes hyperforin and adhyperforin, and the latter, hypericin and pseudohypericin (Figure 5).

Eckert *et al.* [113] studied the effect of hyperforin on crude brain membranes from guinea pigs by measuring FA with DPH and TMA-DPH. They found that hyperforin fluidizes the hydrocarbon cores of brain membranes at 10 µM, but rigidifies the hydrophilic regions. They also showed that the oral administrations of St. John’s wort extract and its component hyperforin result in significant levels of hyperforin in murine brain, which decrease the annular and bulk fluidity of neuronal membranes [114]. Chaloupka *et al.* [115] reported that hypericin also interacts with DMPC liposomal membranes to decrease their fluidity at 3 µM by a microspectrofluorimetric study.

The membrane interactions of hyperforin and hypericin are considered to mechanistically contribute to antidepressant and other neuropharmacological effects of St. John’s wort [114,116].

### 3.6. Organosulfur Compounds

**Figure 6:**
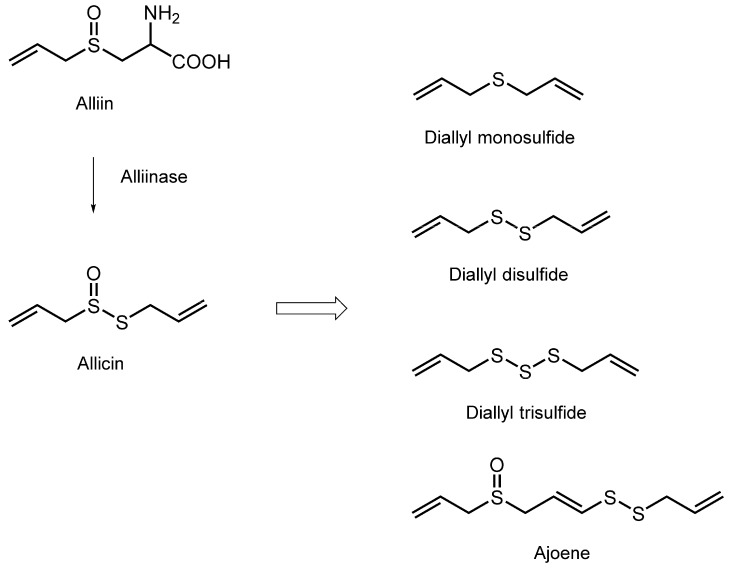
Structures of membrane-interactive organosulfur compounds.

Spice and food additive garlic (*Allium sativum*) has been used for a long time as a medicinal herb because of its health benefits such as cardiovascular, antihypertensive, antiatherosclerotic, antithrombotic, hypolipidemic, antimicrobial, cancer-preventive, immunomodulatory, hepatoprotective, and antidiabetic effects, almost all of which are supported by epidemiological, experimental, and preclinical studies. Garlic and its related plants (onion, shallot, leek, scallion, and chive) contain two classes of bioactive components, flavonoids and organosulfur compounds. The latter phytochemicals provide these plants, especially garlic, with not only a characteristic odor and pungent taste but also various bioactivities. Intact garlic bulbs contain a large amount of γ-glutamyl-*S*-allyl-l-cysteine, which is hydrolyzed and oxidized to form alliin. When the bulbs are crushed, chopped, or damaged, alliinase is released to transform alliin to allicin enzymatically. Allicin is so unstable that it is rapidly converted to diallyl monosulfide, diallyl disulfide, diallyl trisulfide, and ajoene (Figure 6).

Tsuchiya and Nagayama [117] treated biomimetic membranes consisting of varying compositions of DPPC, POPC, POPE, SOPS, sphingomyelin, and cholesterol with allyl derivatives, and then measured FP with DPH, TMA-DPH, 2-AS, 6-AS, 9-AS, 12-AS, and 16-AP. They found that allyl sulfides decrease the fluidity of tumor cell mimetic and platelet mimetic membranes at 20–500 μM with the potency being diallyl trisulfide > diallyl disulfide, both of which preferentially act on the hydrocarbon cores of lipid bilayers. They also revealed the lipid composition-dependent membrane effects that allyl sulfides rigidify at 100–500 μM *Candida* cell mimetic membranes prepared with ergosterol to show the relative potency of diallyl disulfide > diallyl trisulfide > diallyl monosulfide, but not bacterial cell mimetic membranes without ergosterol, being consistent with the specific antifungal effects of allyl sulfides. Unlike membrane-interactive allyl sulfides, however, their precursor alliin is not effective in interacting with any membranes. In contrast to membrane-rigidifying allyl sulfides, Debouzy *et al.* [118] showed that ajoene increases the fluidity of hydrocarbon chains of unilamellar vesicles prepared with phospholipids and cholesterol by an ESR spectroscopic study. Rendu *et al.* [119] reported the similar ESR spectroscopic results that ajoene decreases the microviscosity of platelet plasma membranes and artificial lipid membranes by acting on the internal regions of lipid bilayers, but not affecting the outer hydrophilic moieties.

The antimicrobial, antitumor, and antiplatelet effects of garlic organosulfur components are related to their membrane interactions [117,120].

### 3.7. Alkaloids

Several alkaloids have the properties to interact with artificial and biological membranes to change the fluidity in association with their pharmacological effects [121]. Representative membrane-interactive alkaloids are β-carboline (pyrido[3,4-*b*]indole) alkaloids, opium alkaloids, piperidine alkaloids, and isoquinoline alkaloids.

**Figure 7:**
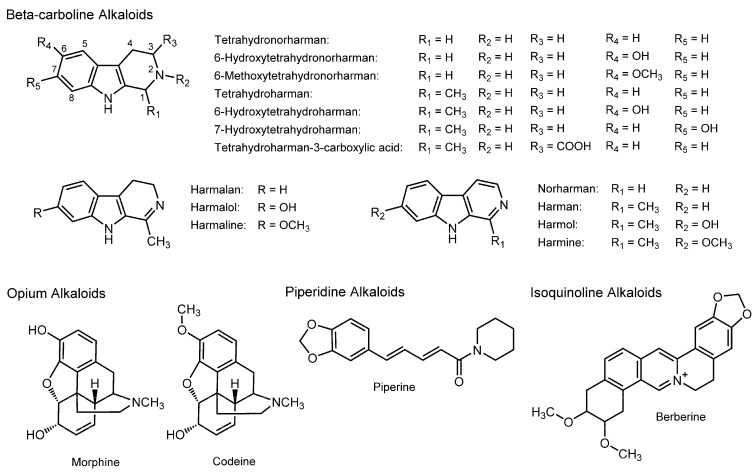
Structures of membrane-interactive alkaloids.

The general term “passionflower” is used for various species of the genus *Passiflora*, of which *Passiflora incarnata* is the most popular herb with a medicinal value, followed by *Passiflora edulis* and *Passiflora foetida*. Leaves, roots, and tea products of passionflower have been used for insomnia, anxiety, epilepsy, neuralgia, hysteria, ulcers, and burns. *Passiflora incarnata* and *Passiflora foetida* are also known to have anxiolytic, analgesic, and anti-inflammatory effects. Bioactive components in these plants are flavonoids (such as apigenin, luteolin, quercetin, kaempferol, and chrysin) and alkaloids. The relevant alkaloids include β-carbolines harman (1-methyl-β-carboline), norharman (β-carboline), harmol (7-hydroxy-1-methyl-β-carboline), and harmine (7-methoxy-1-methyl-β-carboline); dihydro-β-carbolines harmalan (1-methyl-3,4-dihydro-β-carboline), harmalol (7-hydroxy-1-methyl-3,4-dihydro-β-carboline), and harmaline (7-methoxy-1-methyl-3,4-dihydro-β-carboline); and tetrahydro-β-carbolines tetrahydroharman (1-methyl-1,2,3,4-tetrahydro-β-carboline), tetrahydronorharman (1,2,3,4-tetrahydro-β-carboline), and tetrahydroharmol (7-hydroxy-1-methyl-1,2,3,4-tetrahydro-β-carboline) (Figure 7). Beta-carboline alkaloids were originally isolated from *Peganum harmala*, which has been traditionally used for ritual and medicinal preparations in the Middle East, North Africa, and Central Asia [122]. Of interest, β-carboline alkaloids are not only distributed in medicinal plants, herbs, fruits, and vegetables, but are also found in human tissues and body fluids. Different β-carboline alkaloids show the inhibitory effect on amine neurotransmitter metabolism and the affinity for serotonin, dopamine, benzodiazepine, and imidazoline receptors in the central nervous system. They also have cardiovascular (vasorelaxant, antihypertensive, and antithrombotic), analgesic, antinociceptive, antidepressive, antimicrobial, antiviral, antiparasitic, DNA intercalating, and hallucinogenic properties [123].

Peura *et al.* [124] treated DPPC liposomes with tetrahydro-β-carbolines and determined their induced changes in membrane fluidity by FP measurements with PNA and ANS. They found that tetrahydroharman and tetrahydronorharman increase the membrane fluidity at high micromolar concentrations. Tsuchiya and Ohmoto [125] studied the membrane interactions of various β-carboline alkaloids relating to their antiplatelet effects. They showed that β-carbolines (harman, norharman, harmol, and harmine), dihydro-β-carbolines (harmalan, harmalol, and harmaline), and tetrahydro-β-carbolines (tetrahydroharman, tetrahydronorharman, 6-hydroxytetrahydroharman, and 7-hydroxytetrahydroharman (tetrahydroharmol)) differently influence platelet aggregation induced by collagen, epinephrine, ADP, PAF, and thrombin and that the most potent tetrahydroharman inhibits the aggregation at 15–177 μM by up to 50%. They also revealed that antiplatelet tetrahydroharman increases the fluidity of platelet mimetic membranes composed of POPC and cholesterol at platelet aggregation-inhibitory concentrations by measuring FP with PNA and ANS. By measuring FP with PNA, ANS, DPH, and TMA-DPH, Tsuchiya [126] demonstrated that tetrahydroharman has a concentration-dependent biphasic effect to decrease the fluidity of the inner layers of POPC and cholesterol liposomes at low nanomolar concentrations but increase it at high micromolar concentrations. However, the membrane interactivity of tetrahydroharman is significantly reduced or lost by hydroxylation at the 6- or 7-position as reported for tetrahydroharmol [127]. Membrane-rigidifying tetrahydro-β-carbolines could counteract the membrane fluidization by general and local anesthetics to affect their anesthetic effects [127,128].

Membrane-interactive β-carboline alkaloids are referred to as one of the phytochemicals responsible for the neuropharmacological and antiplatelet effects of the plants belonging to the genera *Passiflora* and *Peganum* [125].

Morphine and its relating opium alkaloids (Figure 7), originating from the opium poppy (*Papaver somniferum*), act on the central nervous system to induce analgesia, sedation, cough reflex suppression, respiratory depression, and nausea. They are generally recognized to exert analgesic and sedative effects by binding to opioid receptors (μ-, κ-, and δ-opioid receptors). In addition, opium alkaloids are able to interact with lipid membranes and affect their physicochemical properties. By Fourier transform infrared spectroscopic and fluorescence depolarization experiments using rat models of morphine dependence, Nie *et al.* [45] showed that morphine significantly decreases the fluidity of erythrocyte membranes by directly acting on membrane lipids, not through opioid receptors. Budai *et al.* [129] reported the DSC and EPR spectroscopic studies in which analgesic morphine, antitussive codeine, and their derivatives act on DPPC unilamellar vesicles to decrease the mobility of the polar head groups.

Piperidine alkaloids include piperine (Figure 7), a pungent component in black pepper (*Piper nigrum*) and long pepper (*Piper longum*) that are used as spices and food preservatives, and for the medicinal purpose of enhancing digestive capacity and protecting against oxidative damage. Since it also possesses the bioavailability-enhancing activity of other drugs, Khajuria *et al.* [130] studied *in vivo* and *in vitro* membrane effects of piperine by fluorospectroscopy with pyrene. They found that the fluidity of intestinal brush-border membranes is increased 5–15 min after the oral administration of piperine (5–20 mg/kg body weight) to rats and that membrane fluidity is also increased by treating the brush-border membrane vesicles from rat jejunum with 2–50 μM piperine. Prakash and Srinivasan [109] maintained rats on diets containing 0.02% piperine for eight weeks and determined the fluidity of intestinal brush-border membranes by measuring FP with DPH. They showed that piperine increases the membrane fluidity in ileum and jejunum, and stimulates the activities of different enzymes in small intestinal mucosa, suggesting that the drug bioavailability-enhancing property of piperine is attributable to the increased absorption due to changes of membrane lipid dynamics and intestinal enzyme conformation.

Berberine is one of isoquinoline alkaloids (Figure 7) present in such plant species as *Berberis vulgaris*, *Berberis aristata*, *Berberis aquifolium*, and *Hydrastis canadensis*, which have been considered to possess anti-inflammatory, antimicrobial, antiproliferative, and apoptosis-inducing effects. Gąsiorowska *et al.* [131] studied the membrane interaction of berberine by DSC and FP experiments. They found that berberine acts on negatively charged DMPG unilamellar vesicles to reduce the phospholipid phase transition temperature.

### 3.8. Anthraquinonoids

**Figure 8:**
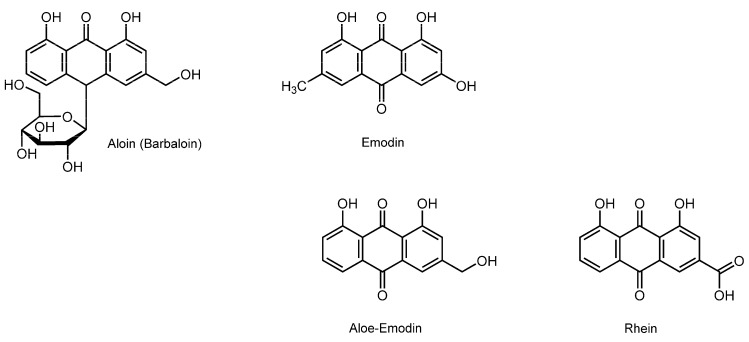
Structures of membrane-interactive anthraquinonoids.

Aloe, the “plant of immortality”, belongs to the genus comprising over 500 species. The most widely known species is *Aloe vera*, followed by *Aloe ferox* and *Aloe arborescens*. These aloe plants have been traditionally used for diabetes, burns, osteoarthritis, inflammatory diseases, asthma, and epilepsy. They contain anthraquinonoids, a class of phenolic phytochemicals with a 9,10-anthraquinone skeleton, such as aloin (barbaloin), emodin, aloe-emodin, rhein, chrysophanol, and danthron (Figure 8).

Alves *et al.* [132,133] showed that aloin and emodin interact with DMPC or DMPG unilamellar vesicles to perturb the membrane property and structure by DSC and ESR spectroscopic studies. The membrane interactions of its anthraquinonoid components are responsible for diverse bioactivities of aloe.

### 3.9. Ginsenosides

**Figure 9:**
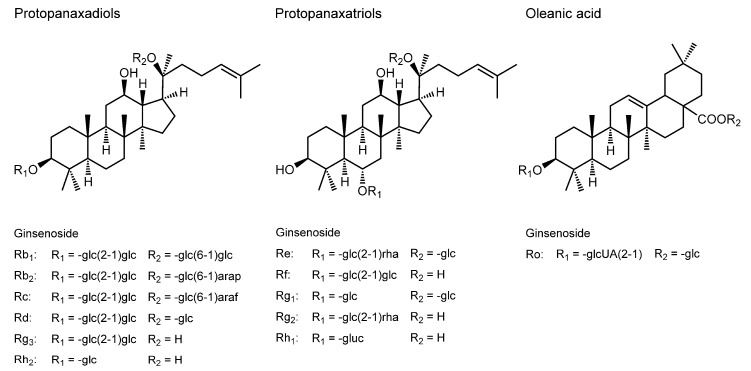
Structures of membrane-interactive ginsenosides.

While the dried roots of ginseng have established a permanent position as folk medicine or ethnomedicine in Asian countries, in recent years, ginseng preparations have been gaining popularity in Western countries. Ginseng has been traditionally considered to possess general health-promoting effects on the central nervous, cardiovascular, immune, and endocrine systems, and antineoplastic, antioxidant, antidiabetic, and anti-inflammatory potentials. Three common species of ginseng are *Panax ginseng* (Asian ginseng), *Panax quinquefolius* (American ginseng), and *Panax japonicus* (Japanese ginseng). The herbal products of *Panax ginseng* are classified by the difference in its processing. “Fresh” is a raw product, “red” is a product dried after steaming, and “white” is a product dried without heating. Ginsenosides are the primary bioactive components of ginseng. Ginsenosides Rb_1_, Rb_2_, Rc, Re, Rf, Rg_1_, and Rg_2_ originate from “fresh” and “white” ginseng, and ginsenoside Rg_3_, Rg_5_, Rh_1_, and Rh_2_, from “red” ginseng. Ginsenoside, known as ginseng saponin, belongs to triterpene glycosides as described in Section 3.2. They may also be referred to as phytosterols because ginsenosides, except for ginsenoside Ro, have the steroidal structure. Based on the structure of aglycone, ginsenosides are divided into protopanaxadiols (ginsenoside Rb_1_, Rb_2_, Rc, Rd, Rg_3_, and Rh_2_), protopanaxatriols (ginsenoside Re, Rf, Rg_1_, Rg_2_, and Rh_1_), and oleanic acid (ginsenoside Ro) (Figure 9).

By using two-photon fluorescence microscopy and generalized polarization imaging, Yi *et al.* [134] demonstrated that ginsenosides Rb_2_, Rc, Rd, Re, Rf, Rg_1_, Rg_2_, and Rh_2_ increase the membrane fluidity of HeLa cells at 50 µM and disrupt lipid rafts that are the membrane platforms to mediate the signal transduction pathways for cell apoptosis and proliferation. Among them, ginsenoside Rh_2_ was speculated to cause ligand-independent Fas activation via the lipid raft disruption. Zhou *et al.* [135] studied the membrane interaction of ginsenoside Re by measuring FP with DPH and related it to the protective effect against cerebral ischemia/reperfusion injuries in rats. They found that the fluidity of brain mitochondrial membranes is increased after the oral administration of ginsenoside Re (5–20 mg/kg body weight) to rats once a day for seven days. In contrast, Kwon *et al.* [136] reported that ginsenoside Rg_3_ decreases the membrane fluidity of human fibroblast carcinoma cells at 20 µM, but not the counterpart normal cells, by FA measurements with DPH and TMA-DPH. Tachikawa *et al.* [137] also showed that ginsenoside Rg_3_, but not Rg_2_, increases the membrane microviscosity of bovine adrenal chromaffin cells at 30 μM by measuring FA with DPH.

The membrane interactions of ginsenosides are at least partly responsible for the protective effects of ginseng against ischemia-reperfusion injuries and tumor cells [135,136].

### 3.10. Pentacyclic Triterpene Acids

**Figure 10:**
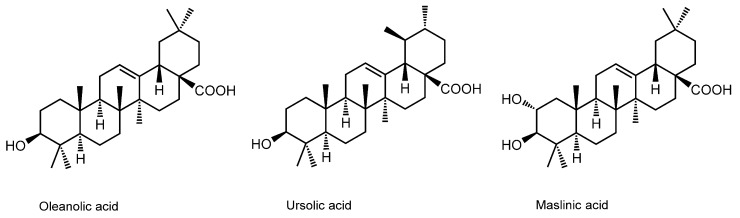
Structures of membrane-interactive pentacyclic triterpene acids.

Cranberries, bilberries, and blueberries are the fruits obtained from shrubs belonging to the genus *Vaccinium*, including *Vaccinium oxycoccos* (northern cranberry), *Vaccinium myrtillus* (bilberry or European blueberry), and *Vaccinium angustifolium* (lowbush blueberry). Their juice, extracts, and leaves have been known as folk medicines for urinary tract infection, eye conditions (cataracts, glaucoma, and retina disorders), inflammatory diseases, cardiovascular diseases (hypertension and hypercholesterolemia), and antioxidant cancer prevention. Besides flavonoids (such as luteolin, rutin, quercetin, myricetin, EGC, EGCG, cyanidin, and delphinidin) and stilbenoids (such as resveratrol and pterostilbene), bioactive pentacyclic triterpene acids oleanolic (oleanic) acid, ursolic acid, and maslinic acid are contained in *Vaccinium* berries (Figure 10).

Han *et al.* [138] treated DPPC liposomal membranes with pentacyclic triterpene acids, and then measured FP with DPH. They found that oleanolic acid and ursolic acid increase membrane fluidity in the crystalline state, but decrease it in the liquid-crystalline state. Broniatowski *et al.* [139] also reported that these pentacyclic triterpene acids target anionic phospholipids and fluidize the model membranes consisting of cardiolipin and phosphatidylglycerol.

### 3.11. Curcuminoids

**Figure 11:**
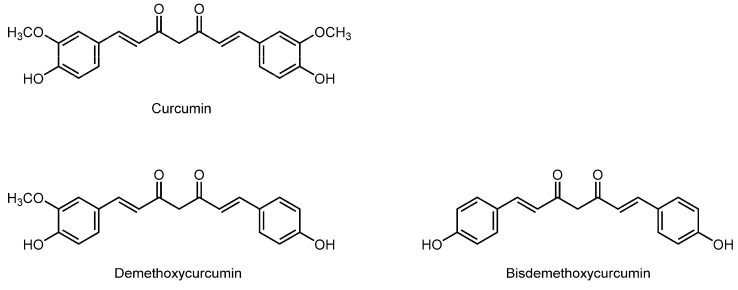
Structures of membrane-interactive curcuminoids.

Curcuminoids comprise a class of polyphenols with the structure of linear diarylheptane. Turmeric (*Curcuma longa*, belonging to the ginger family *Zingiberaceae*) is a primary plant containing curcuminoids to produce a distinct yellow color. Turmeric has been widely used as a spice and also as a folk medicine for conditions such as indigestion, pain, arthritis, and infection. Representative curcuminoids are curcumin ((1*E*,6*E*)-1,7-bis(4-hydroxy-3-methoxyphenyl)-1,6-heptadiene-3,5-dione), demethoxycurcumin, and bisdemethoxycurcumin (Figure 11). The C_7_ chain is di-unsaturated to show an *E*-configuration (*trans* C=C bond) and two aryl rings are substituted with hydroxyl or methoxyl groups. Curcuminoids, especially curcumin, have been attracting medicinal attention for antioxidant, hypolipidemic, antiproliferative, antimicrobial, anti-inflammatory, antidiabetic, cardioprotective, antiatherosclerotic, and antidepressant effects.

Jaruga *et al.* [140] revealed that curcumin acts on human erythrocytes to increase the membrane fluidity at 100–150 μM by an ESR spectroscopic study. Kempaiah and Srinivasan [108] maintained experimental hypercholesterolemic rats on diets containing 0.2% curcumin for eight weeks in order to investigate the molecular mechanism underlying a hypolipidemic effect of curcumin. Their ESR spectroscopy and FA measurements with DPH indicated that curcumin increases the fluidity of rat erythrocyte membranes and reverses the fluidity changes induced in them. Atsumi *et al.* [141] showed that curcumin acts on human submandibular adenocarcinoma cells and primary gingival fibroblasts to lower the membrane mobility at 10 μM. Sun *et al.* [142] reported that curcumin interacts with DOPC unilamellar vesicles to alter the hydrocarbon thickness and the elastic rigidity of lipid bilayers. Hung *et al.* [143] also reported that curcumin decreases the thickness of DOPC bilayer membranes and weakens their elasticity.

The membrane interactions are contributable to hypolipidemic, antitumor, and other beneficial effects of curcuminoids as suggested by previous studies [108,140].

### 3.12. Bioavailability of Membrane-Interactive Phytochemicals

Although different classes of phytochemicals are expected for potential therapeutic use against diseases and preclinical application as drug candidates, a question is raised as to whether they can exhibit the membrane-interacting properties in human and animal bodies because the relevant phytochemicals may be poorly absorbed, efficiently metabolized, and/or rapidly excreted. The limited absorption and stability of phytochemicals prevent them from achieving *in vivo* concentrations high enough to exert significant effects associated with membrane interaction. Poor water-solubility (relatively high hydrophobicity) linked to membrane interactivity is one of the common reasons for the low oral bioavailability of phytochemicals, because the passing through aqueous layers on the surface of intestinal epithelia is essentially required for absorption. Endogenous factors such as intestinal microbiota and digestive enzymes also considerably affect the absorptive and metabolic efficiency of phytochemicals [144]. Among membrane-interactive phytochemicals contained in medicinal plants, herbs, dietary botanical supplements, and functional plant food, flavonoids (flavonols, flavones, and flavanols) and terpenoids (ginsenosides) have been exclusively studied for bioavailability, especially absorption through the gastrointestinal tracts.

Aglycones with significant membrane interactivity can be more easily absorbed from the small intestine compared with glycosides. However, naturally occurring flavonoids are mostly glycosides. Once absorbed, flavonoids undergo first-pass metabolism via conjugation and subsequent excretion, although enteric and enterohepatic recycling possibly allows bioactive flavonoid aglycones to be present *in vivo* for a long time [145]. Flavonoid glycosides are subject to deglycosylation by intestinal bacteria to increase the absorption efficiency, whereas flavonoids are degraded by colonic microflora [146]. The small intestine also acts as an absorption site for quercetin glucoside and other glucose-bound flavonoids because small intestinal cells possess glucoside-hydrolyzing activity [147]. Human pharmacokinetic studies indicate the great oral bioavailability of flavonoids, so that the blood concentrations of quercetin and EGCG are high enough to exert their pharmacological effects after oral administration [148].

Naturally occurring ginsenosides are triterpene glycosides primarily consisting of two different aglycones (protopanaxadiol and protopanaxatriol) which contain several sugars at the C-3 or C-6 and C-20 positions (Figure 9). Deglycosylated ginsenosides show greater bioactivities than glycosylated ones because of a difference in absorption efficiency [149]. Shin *et al.* [150] administered red ginseng extracts (20–2000 mg/kg/day) orally to mice and analyzed the major ginseng components in the blood. Consequently, the blood concentrations of ginsenoside Rb_1_, Rg_1_, and Re were negligible, indicating the poor absorption of ginsenosides in the original form. In an *in vitro* study using a Coca-2 cell monolayer model and oral administration to rats, Han and Fang [151] suggested that the low oral bioavailability of a major ginseng component, ginsenoside Rg_1_, is due to its poor membrane permeability and its elimination in gastrointestinal tracts and liver. In humans and rats, ginsenoside Rg_1_ is metabolically hydrolyzed to different ginsenosides Rg_3_, Rh_2_, and compound K (20-*O*-β-d-glucopyranosyl-20(*S*)-protopanaxadiol) by intestinal microflora. Kim [152] orally administered red ginseng extract (9 g) to human male subjects and analyzed blood samples collected after 4–36 h. The results showed that the mean maximum plasma concentration of compound K is significantly higher than that of ginsenoside Rb_1_, which is accounted for by the intestinal microflora that transforms ginsenoside Rb_1_ to compound K. Although membrane interactivity has not been reported for compound K, this secondary ginsenoside should exert more potent *in vivo* effects than the parent ginsenosides.

## 4. Membrane Interaction Applicable to the Discovery of Phytochemical Drug Leads

While phytochemicals influence numerous cell functions by modulating the activities of membrane-associated proteins, their diverse effects may be due to interactions with cellular membranes rather than specific binding to proteins [153]. Almost all of the membrane-interactive drugs and phytochemicals commonly have the ability to modify fluidity, microviscosity, order, elasticity, and permeability of biological and artificial membranes. They either enhance or reduce these membrane properties, correlating to their pharmacological and toxicological effects. When using plants as starting points for drug development, there are different approaches to selecting the phytochemicals for drug leads. The membrane interaction, especially the resulting fluidity change, is usable as a clue to discover the potential drug leads. The membrane interactivity would be one of the experimental guides for (1) determining the quantitative structure and activity relationship (QSAR) to characterize the phytochemical structure with the greater bioactivity and (2) performing the screening and purification of plant components to isolate the medicinally promising phytochemical.

The QSAR can offer important information to predict the structural requirements for phytochemicals with both membrane interactivity and bioactivity. The QSAR on membrane interaction has been most frequently applied to flavonoids in association with their antioxidant, antimicrobial, antitumor, and antiplatelet effects [154]. Arora *et al.* [72] studied the localization of flavonoids and isoflavonoids in lipid bilayers and their induced changes in membrane fluidity by measuring FA of SLPC unilamellar vesicles with 6-AS, 12-AS, and 16-AP. They found the structure-dependence of these phytochemicals to induce membrane fluidity decreases, which are linked to the antioxidant properties of flavonoids. Erlejman *et al.* [155] compared the effects of 26 flavonoids and their related compounds on membrane fluidity and lipid oxidation. Their characterized QSAR indicated that the antioxidant potencies of flavonoids are determined by membrane interaction to order or rigidify phosphatidylcholine membranes, which depends on the hydrophobicity of flavonoids and the number of hydroxyl groups in their structures. Wu *et al.* [80] examined the antibacterial effects of 11 flavonoids while evaluating their membrane interactions by measuring FP of DPPC and DPPG unilamellar vesicles with DPH. They revealed a positive correlation between antibacterial potency (the minimum concentration to inhibit the growth of 50% of *Escherichia coli*) and membrane interactivity (the decreasing effect on membrane fluidity). Their obtained QSAR suggested that the antibacterial activity is potentiated by the hydrophobicity of membrane-interactive flavonoids and the presence of specific substituents at the 3-position. Sinha *et al.* [156] related the antiproliferative and antioxidant effects of flavonols to their interactions with DPPC bilayer membranes by DSC and NMR spectroscopic experiments. The comparison of galangin, quercetin, and fisetin showed that the number and the position of hydroxyl groups are important to increase both membrane interactivity and bioactivity. Tsuchiya [79,82] comparatively studied the interactions of 32 flavonoids with liposomal membranes consisting of POPC, POPE, POPS (or SOPS), and cholesterol by measuring FP with 2-AS, 6-AS, 9-AS, 12-AS, and 16-AP together with determining the antiproliferative activity. The characterized QSAR indicated that a 3-hydroxyl group and a double bond between 2- and 3-carbon of the C ring, 3′,4′-dihydroxyl groups of the B ring, and 5,7-dihydroxyl groups of the A ring are the determinants to increase both the interaction with membranes and the antiproliferative effect on tumor cells. Cyanidin, quercetin, galangin, and EGCG meeting such structural requirements significantly decreased the membrane fluidity of tumor cells at 10 μM while simultaneously inhibiting their proliferation. Tsuchiya [86,87] also compared eight structurally different flavanols and catechin stereoisomers for the membrane fluidity-decreasing effects on DPPC, DOPC, and POPC/cholesterol liposomes by measuring FP with PNA and ANS. The results indicated that EGCG has the greatest membrane interactivity associated with hepatoprotective and antiplaque activity and that catechins stereostructure-specifically interact with the membranes to show the relative potency of (–)-epicatechin > (+)-epicatechin > (–)-catechin > (+)-catechin in the presence of chiral cholesterol in membranes. Based on the QSAR analysis, EGCG was specified as the most potent phytochemical to decrease the membrane fluidity at 5–100 μM, suggesting the importance of a galloyl group at the 3-position.

Nowotarska *et al.* [91] verified the relation between antibacterial activity and membrane interactivity of terpenoids. Reiner *et al.* [93] correlated the positive modulation of GABA_A_ receptors by monocyclic monoterpenoids with their membrane fluidization of DPPC unilamellar vesicles by measuring FA with DPH and TMA-DPH. Their characterized QSAR indicated that the GABAergic pharmacological activity is potentiated by the structural lipophilicity of monoterpenoids.

Tsuchiya and Ohmoto [125] compared the antiplatelet effects of β-carboline alkaloids and their interactions with platelet mimetic membranes. Based on the FP decreases measured with ANS and PNA, they showed that tetrahydroharman is the most potent alkaloid to inhibit human platelet aggregation induced by different stimulants (collagen, ADP, epinephrine, PAF, and thrombin) and increase the membrane fluidity by acting on the superficial and deeper regions of lipid bilayers. In their determined QSAR, both membrane interactivity and antiplatelet activity were significantly decreased or lost by the structural modifications such as 6-hydroxylation, 7-hydroxylation, 3-carboxylation, demethylation, and oxidation.

As shown by previous studies, the QSAR on membrane interaction is useful for the structural specification of bioactive phytochemicals to be possible drug leads [157]. By using the membrane interaction as an experimental index, several plants have been successfully screened and fractionated to isolate phytochemicals with medicinal potentials and identify their molecular structures. Furusawa *et al.* [158] carried out a series of extraction and chromatographic fractionation of onion specimens to evaluate the bioactivity and the membrane interactivity of obtained extracts and fractions. Consequently, they isolated antiplatelet phytochemicals, quercetin dimers, to inhibit human platelet aggregation induced by collagen and ADP in association with membrane interaction to decrease the fluidity of platelet mimetic membranes. They also revealed that such membrane-interactive flavonoids effectively inhibit the proliferation of tumor cells [159]. Tsuchiya *et al.* [160] applied the membrane interactivity-guided extraction and chromatography to edible wild plant *Evodiopanax innovans*. They found that methanol extracts and specified fractions decrease the fluidity of biomimetic phospholipids/cholesterol membranes. They finally isolated a membrane-rigidifying phytochemical and identified it as maltol 3-*O*-β-glucopyranoside which is able to inhibit both tumor cell growth and platelet aggregation. Oyedapo *et al.* [161] examined the activities of plant extracts and fractions to stabilize erythrocyte membranes relating to their anti-inflammatory effects. The interactions with human and bovine erythrocyte membranes were also applied to the screening of plant species with fruitful results to verify anti-inflammatory and antioxidant effects [162,163].

## 5. Conclusions

Phytochemicals as the bioactive substances in medicinal plants commonly interact with biological and artificial biomimetic membranes to modify membrane physicochemical properties, being consistent with their pharmacological features. Such a membrane interaction would be useful for discovering drug leads from plant resources based on a novel mechanistic point of view of phytochemicals. The promising effects of specified phytochemicals may cover antitumor, antiplatelet, antioxidant, antimicrobial, anti-inflammatory, analgesic, and anesthetic ones, as well as membrane-interactive drugs. The membrane interactions of phytochemicals to modify the physicochemical properties of membranes are summarized in Table 1.

**Table 1:** Phytochemicals with the membrane interactivity.

Phytochemicals or Plants	Used Membranes	Experimental Methods	Membrane Effects	References
**Flavonoids**				
Naringenin; Rutin	SLPC unilamellar vesicles	FA with 6-AS, 12-AS and 16-AP	Decrease the fluidity	[72]
Apigenin; Luteolin; Kaempferol; Quercetin; Myricetin; Naringenin; Eriodictyol; Taxifolin; Pelargonidin; Cyanidin; Delphinidin; (–)-Epicatechin; (–)-Epigallocatechin; (–)-Epigallocatechin-3-gallate; Phloretin	POPC/POPE/POPS/cholesterol unilamellar vesicles	FP with 2-AS, 6-AS, 9-AS, 12-AS and 16-AP	Decrease the fluidity	[79]
Kaempferol; Quercetin; Chrysin; Baicalein; Luteolin	DPPC unilamellar vesicles; DPPG unilamellar vesicles	FP with DPH	Decrease the fluidity	[80]
Galangin; Quercetin; Kaempferol; Chrysin; Baicalein; Fisetin; Luteolin; Apigenin; Myricetin; Morin; Rutin; Isoquercitrin	POPC/POPE/SOPS/cholesterol unilamellar vesicles	FP with 2-AS, 6-AS, 9-AS, 12-AS and 16-AP	Decrease the fluidity	[82]
Quercetin; (–)-Epigallocatechin-3-gallate	Human U937 monocyte membranes; Jurkat T lymphoblast membranes	FA with TMA-DPH	Decrease the fluidity	[83]
(–)-Epicatechin; (–)-Epigallocatechin; (–)-Epigallocatechin-3-gallate; (–)-Epicatechin-3-gallate	DPPC liposomes; DOPC liposomes	FP with PNA and ANS	Decrease the fluidity	[86]
(–)-Epicatechin; (+)-Epicatechin; (–)-Catechin; (+)-Catechin	DPPC liposomes; DOPC liposomes; DOPC/cholesterol liposomes	FP with PNA and ANS	Decrease the fluidity	[87]
**Isoflavonoids**				
Genistein	SLPC unilamellar vesicles	FA with 6-AS, 12-AS and 16-AP	Decrease the fluidity	[72]
Puerarin; Ononin; Daidzein; Genistin	DPPC unilamellar vesicles; DPPG unilamellar vesicles	FP with DPH	Increase the fluidity	[80]
Genistein	Erythrocyte membranes	EPR	Decrease the fluidity	[81]
Daidzein	Erythrocyte membranes	EPR	Increase the fluidity	[81]
**Terpenoids**				
Geraniol	*Candida albicans* cell membranes; DPPC multilamellar vesicles	FA with 2-AS, 6-AS, 9-AS, 12-AS, DPH and TMA-DPH	Increase the fluidity from the membrane surface to the membrane interior	[90]
Carvacrol	Langmuir monolayer membranes of bacterial phospholipids	Surface pressure-area (π-A) and surface potential-area (Δψ-A) isotherms	Increase the fluidity	[91]
Thymol; Eugenol; Carvacrol	DPPC multilamellar vesicles	FA with DPH and TMA-DPH	Increase the fluidity	[93]
Nerolidol; Menthol; Pulegone; Carvone; (+)-Limonene; α-Terpineol; 1,8-Cineol	Mouse fibroblast membranes; Human erythrocyte membranes	EPR	Increase the fluidity	[94]
**Terpenoids**				
Linalool; 1,8-Cineol; α-Terpineol	*Staphylococcus aureus* membranes; *Escherichia coli* membranes	Scanning electron microscopy	Alter the permeability and function	[95]
Citral	Soybean phospholipid liposomes	FP with DPH	Decrease the fluidity	[96]
Borneol	DPPC liposomes	Coarse-grained molecular dynamics simulations	Increase the fluidity	[97]
**Stilbenoids**				
Resveratrol	DPPC liposomes; POPC liposomes; POPC/cholesterol liposomes	FP with DPH and TMA-DPH	Decrease the fluidity	[101]
Resveratrol	Lipid bilayer liposomal membranes	NMR spectroscopy	Decrease the fluidity	[102]
Resveratrol	DPPC unilamellar vesicles	FA with TMA-DPH	Increase the fluidity	[103]
Resveratrol; Piceatannol	DPPC liposomes; DMPC liposomes	DSC; ESR; Fluorescence spectroscopy	Alter the phospholipid phase transition	[104]
**Capsaicinoids**				
Capsaicin	Non-neuronal plasma membranes	FP with DPH and TMA-DPH	Increase the fluidity	[106]
Capsaicin; *N*-Vanillylnonanamide	Platelet mimetic membranes; Bacterial cell mimetic membranes	FP with PNA, DPH and TMA-DPH	Increase the fluidity at ~50 μM Decrease the fluidity at 100–500 μM	[107]
Maintaining rats on diets containing 0.015% capsaicin for eight weeks	Rat erythrocyte membranes	ESR; FA with DPH	Increase the fluidity	[108]
Maintaining rats on diets containing 3.0% chili pepper or 0.01% capsaicin for eight weeks	Rat intestinal brush-border membranes	FP with DPH	Increase the fluidity	[109]
**Phloroglucinols**				
Hyperforin	Guinea pig crude brain membranes	FA with DPH and TMA-DPH	Increase the fluidity of the hydrocarbon cores	[113]
Hyperforin	Guinea pig crude brain membranes	FA with DPH and TMA-DPH	Decrease the fluidity of the hydrophilic regions	[113]
Oral administration of St. John’s wort extract or hyperforin to murine	Murine brain neuronal membranes	FA with DPH and TMA-DPH	Decrease the fluidity	[114]
**Naphthodianthrones**				
Hypericin	DMPC liposomes	Microspectrofluorimetry	Decrease the fluidity	[115]
**Organosulfur Compounds**				
Diallyl trisulfide; Diallyl disulfide	Tumor cell mimetic membranes; Platelet mimetic membranes	FP with DPH, TMA-DPH, 2-AS, 6-AS, 9-AS, 12-AS and 16-AP	Decrease the fluidity by preferentially acting on the hydrocarbon cores	[117]
Diallyl trisulfide; Diallyl disulfide; Diallyl monosulfide	Candida cell mimetic membranes containing ergosterol	FP with DPH, TMA-DPH, 2-AS, 6-AS, 9-AS, 12-AS and 16-AP	Decrease the fluidity	[117]
Ajoene	Phospholipid/cholesterol unilamellar vesicles	ESR	Increase the fluidity of the hydrocarbon chains	[118]
Ajoene	Platelet plasma membranes; Artificial lipid membranes	ESR	Decrease the microviscosity	[119]
**Beta-carboline Alkaloids**				
Tetrahydroharman	Platelet mimetic membranes	FP with PNA and ANS	Increase the fluidity at antiplatelet concentrations	[124]
Tetrahydroharman; Tetrahydronorharman	DPPC liposomes	FP with PNA and ANS	Increase the fluidity at high micromolar concentrations	[125]
Tetrahydroharman	POPC/cholesterol liposomes	FP with PNA, ANS, DPH and TMA-DPH	Decrease the fluidity at low nanomolar concentrations Increase the fluidity at high micromolar concentrations	[126,127,128]
**Opium Alkaloids**				
Morphine	Rat erythrocyte membranes	Fourier transformed infrared spectroscopy; Fluorescence depolarization	Decrease the fluidity	[45]
Morphine; Codeine	DPPC unilamellar vesicles	DSC; EPR	Decrease the mobility of the polar head groups	[129]
Oral administration of piperine (5–20 mg/kg body weight) to rats for 5–15 min	Rat intestinal brush-border membranes	Fluorospectroscopy with pyrene	Increase the fluidity	[130]
Piperine (2–50 μM)	Brush-border membrane vesicles from rat jejunum	Fluorospectroscopy with pyrene	Increase the fluidity	[130]
Maintaining rats on diets containing 0.02% piperine for eight weeks	Rat intestinal brush-border membranes	FP with DPH	Increase the fluidity	[109]
**Anthraquinonoids**				
Aloin; Emodin	DMPC unilamellar vesicles; DMPG unilamellar vesicles	DSC; ESR	Perturb the membrane property and structure	[132,133]
**Ginsenosides**				
Ginsenoside Rb_2_; Rc; Rd; Re; Rf; Rg_1_; Rg_2_; Rh_2_	HeLa cell membranes	Two-photon fluorescence microscopy; Generalized polarization imaging	Increase the fluidity	[134]
Oral administration of ginsenoside Re (5–20 mg/kg body weight) to rats for seven days	Rat brain mitochondrial membranes	FP with DPH	Increase the fluidity	[135]
Ginsenoside Rg_3_ (20 μM)	Human fibroblast carcinoma cell membranes	FA with DPH and TMA-DPH	Decrease the fluidity	[136]
**Pentacyclic Triterpene Acids**				
Oleanolic acid; Ursolic acid	DPPC liposomes	FP with DPH	Increase the fluidity in crystalline state Decrease the fluidity in liquid-crystalline state	[138]
**Curcuminoids**				
Curcumin (100–150 μM)	Human erythrocyte membranes	ESR	Increase the fluidity	[140]
Maintaining experimental hypercholesterolemic rats on diets containing 0.2% curcumin for eight weeks	Rat erythrocyte membranes	ESR; FA with DPH	Increase the fluidity	[108]
Curcumin	DOPC unilamellar vesicles	X-ray diffraction	Thin the lipid bilayer membranes and weaken their elasticity	[143]

## Abbreviations

**ADP:** Adenosine 5′-diphosphate

**ANS:** 1-Anilinonaphthalene-8-sulfonic acid

**16-AP:** 16-(9-Anthroyloxy)palmitic acid

**2-AS:** 2-(9-Anthroyloxy)stearic acid

**6-AS:** 6-(9-Anthroyloxy)stearic acid

**9-AS:** 9-(9-Anthroyloxy)stearic acid

**12-AS:** 12-(9-Anthroyloxy)stearic acid

**COX:** Cyclooxygenase

**DMPC:** 1,2-Dimyristoyl-*sn*-glycero-3-phosphocholine

**DMPG:** 1,2-Dimyristoyl-*sn*-glycero-3-phosphoglycerol

**DOPC:** 1,2-Dioleoyl-*sn*-glycero-3-phosphocholine

**DPH:** 1,6-Diphenyl-1,3,5-hexatriene

**DPPC:** 1,2-Dipalmitoyl-*sn*-glycero-3-phosphocholine

**DPPE:** 1,2-Dipalmitoyl-*sn*-glycero-3-phosphoethanolamine

**DPPG:** 1,2-Dipalmitoyl-*sn*-glycero-3-phosphoglycerol

**DSC:** Differential scanning calorimetry

**EGC:** (–)-Epigallocatechin

**EGCG:** (–)-Epigallocatechin-3-gallate

**EPR:** Electron paramagnetic resonance

**ESR:** Electron spin resonance

**FA:** Fluorescence anisotropy

**FP:** Fluorescence polarization

**GABA_A_:** γ-Aminobutyric acid type-A

**NMDA:***N*-Methyl-d-aspartate

**NMR:** Nuclear magnetic resonance

**NSAIDs:** Non-steroidal anti-inflammatory drugs

**PAF:** Platelet-activating factor

**PNA:***N*-Phenyl-1-naphthylamine

**POPC:** 1-Palmitoyl-2-oleoyl-*sn*-glycero-3-phosphocholine

**POPE:** 1-Palmitoyl-2-oleoyl-*sn*-glycero-3-phosphoethanolamine

**POPS:** 1-Palmitoyl-2-oleoyl-*sn*-glycero-3-[phospho-L-serine]

**QSAR:** Quantitative structure and activity relationship

**QX-314:***N*-(2,6-Dimethylphenylcarbamoylmethyl)triethylammonium bromide

**SLPC:** 1-Stearoyl-2-linoleoyl-*sn*-glycero-3-phosphocholine

**SOPS:** 1-Stearoyl-2-oleoyl-*sn*-glycero-3-[phospho-L-serine]

**TMA-DPH:** 1-4-(Trimethylamino)phenyl]-6-phenyl-1,3,5-hexatriene

**TRPV1:** Transient receptor potential vanilloid type-1
